# Rhabdomyolysis, Acute Kidney Injury, and Mortality in Ebola Virus Disease: Retrospective Analysis of Cases From the Eastern Democratic Republic of the Congo, 2019

**DOI:** 10.1093/infdis/jiae224

**Published:** 2024-05-02

**Authors:** Masumbuko Claude Kasereka, Daniel Mukadi-Bamuleka, Richard Kitenge-Omasumbu, François Edidi-Atani, Meris Matondo Kuamfumu, Sabue Mulangu, Olivier Tshiani-Mbaya, Kambale Malengera Vicky, Placide Mbala-Kingebeni, Steve Ahuka-Mundeke, Jean-Jacques Muyembe-Tamfum, Bonita E Lee, Stan Houston, Zubia Mumtaz, Michael T Hawkes

**Affiliations:** Department of Medicine, Université Catholique du Graben, Butembo, Democratic Republic of the Congo; School of Public Health, University of Alberta, Edmonton, Canada; Department of Virology, Institut National de Recherche Biomédicale; Service of Microbiology, Department of Medical Biology, University of Kinshasa; Programme National d’Urgences et Actions Humanitaires, Ministry of Health of the Democratic Republic of the Congo, Kinshasa; Department of Virology, Institut National de Recherche Biomédicale; Service of Microbiology, Department of Medical Biology, University of Kinshasa; Department of Virology, Institut National de Recherche Biomédicale; Service of Microbiology, Department of Medical Biology, University of Kinshasa; Department of Virology, Institut National de Recherche Biomédicale; Service of Microbiology, Department of Medical Biology, University of Kinshasa; Department of Virology, Institut National de Recherche Biomédicale; Service of Microbiology, Department of Medical Biology, University of Kinshasa; Department of Medicine, Université Catholique du Graben, Butembo, Democratic Republic of the Congo; Department of Virology, Institut National de Recherche Biomédicale; Service of Microbiology, Department of Medical Biology, University of Kinshasa; Department of Virology, Institut National de Recherche Biomédicale; Service of Microbiology, Department of Medical Biology, University of Kinshasa; Department of Virology, Institut National de Recherche Biomédicale; Service of Microbiology, Department of Medical Biology, University of Kinshasa; Department of Pediatrics, University of Alberta, Edmonton; School of Public Health, University of Alberta, Edmonton, Canada; School of Public Health, University of Alberta, Edmonton, Canada; School of Public Health, University of Alberta, Edmonton, Canada; Department of Pediatrics, University of British Columbia, Vancouver, Canada

**Keywords:** acute kidney injury, Ebola virus disease, mortality, rhabdomyolysis, Democratic Republic of the Congo

## Abstract

**Background:**

Skeletal muscle injury in Ebola virus disease (EVD) has been reported, but its association with morbidity and mortality remains poorly defined.

**Methods:**

This retrospective study included patients admitted to 2 EVD treatment units over an 8-month period in 2019 during an EVD epidemic in the Democratic Republic of the Congo.

**Results:**

An overall 333 patients (median age, 30 years; 58% female) had at least 1 creatine kinase (CK) measurement (n = 2229; median, 5/patient [IQR, 1–11]). Among patients, 271 (81%) had an elevated CK level (>380 U/L); 202 (61%) had rhabdomyolysis (CK >1000 IU/L); and 45 (14%) had severe rhabdomyolysis (≥5000 U/L). Among survivors, the maximum CK level was a median 1600 (IQR, 550–3400), peaking 3.4 days after admission (IQR, 2.3–5.5) and decreasing thereafter. Among fatal cases, the CK rose monotonically until death, with a median maximum CK level of 2900 U/L (IQR, 1500–4900). Rhabdomyolysis at admission was an independent predictor of acute kidney injury (adjusted odds ratio, 2.2 [95% CI, 1.2–3.8]; *P* = .0065) and mortality (adjusted hazard ratio, 1.7 [95% CI, 1.03–2.9]; *P* = .037).

**Conclusions:**

Rhabdomyolysis is associated with acute kidney injury and mortality in patients with EVD. These findings may inform clinical practice by identifying laboratory monitoring priorities and highlighting the importance of fluid management.

## Teaser

In this retrospective of 333 patients with Ebola virus disease (Democratic Republic of the Congo, 2019), rhabdomyolysis at admission was an independent predictor of acute kidney injury (adjusted odds ratio, 2.2 [95% CI, 1.2–3.8]; *P* = .0065) and mortality (adjusted hazard ratio, 1.7 [95% CI, 1.03–2.9]; *P* = .037).

Ebola virus disease (EVD) is a highly contagious and severe febrile illness [1] caused by Ebola virus (EBOV; *Orthoebolavirus zairense* spp) [2]. EVD case fatality rates range from 25% to 90% [3]. The 2 largest epidemics to date were reported in West Africa from 2013 to 2016 (11 000 deaths) and the eastern Democratic Republic of the Congo (DRC) from 2018 to 2020 (2264 deaths) [4]. Standard-of-care treatment of EVD relies on early administration of specific therapeutics (ie, monoclonal antibodies) as well as aggressive supportive care [1, 3]. Management of EVD cases should be guided by the monitoring of laboratory parameters such as viral load, hematology, and blood chemistry, including creatine kinase (CK) [3].

CK is a cytosolic enzyme in myocytes that catalyzes the conversion of creatine phosphate to creatine and adenosine triphosphate to provide energy for muscle activity. Myocyte injury can result in the release of intracellular elements, including CK, into the extracellular space and bloodstream [5]. Thus, elevated levels of circulating CK are commonly associated with skeletal muscle injury (eg, myositis, muscle necrosis, rhabdomyolysis) [6].

EVD is a multiple-organ dysfunction syndrome that also affects skeletal muscle. EVD is associated with massive cytolysis of several target cells, such as macrophages, fibroblasts, and hepatocytes [7]. Previous studies showed that rhabdomyolysis was common in EVD cases, although these studies were conducted on only small sets of patients [8]. The pathogenesis of rhabdomyolysis in EVD may involve acute damage of the sarcolemma, with the release of myocyte components into the circulation [9]. Free circulating myoglobin can induce acute kidney injury (AKI) through tubular obstruction, direct tubular epithelial injury, or vasoconstriction. In EVD, myoglobin may be one of several causes of acute tubular necrosis that may also include direct viral infection, hypovolemia, or cytokine-mediated nephrotoxicity [3, 10–12].

The objectives of this study were to (1) describe the factors associated with rhabdomyolysis in patients hospitalized with EVD, (2) examine the association between rhabdomyolysis and AKI, and (3) examine the association between rhabdomyolysis and mortality in patients with EVD.

## METHODS

### Study Design and Setting

We conducted a retrospective observational study of patients admitted to the EVD treatment units in Butembo and Katwa, North Kivu, DRC, between March and October 2019. EVD treatment units were temporary facilities used for the isolation and treatment of patients with EVD. Management included administration of a direct-acting antiviral agent: ansuvimab (Ebanga; also known as mAb114 [13]), atoltivimab/maftivimab/odesivimab (Inmazeb; also known as REGN-EB3 [13]), Zmapp [13], or remdesivir. Experimental therapies were administered under the protocols of the MEURI framework (Monitored Emergency Use of Unregistered Interventions) or PALM clinical trial (Pamoja Tulinde Maisha [13]). Additional management of cases consisted of antibiotics, antimalarials, parenteral fluids, glucose, and electrolyte replacement, as clinically indicated.

### Laboratory Methods

Plasma or whole blood chemistry values, including CK, were determined by the Piccolo AmLyte13 disc run on the Piccolo Xpress Chemistry Analyzer (Abaxis). The principle of quantitative detection of CK involves the enzymatic conversion by CK of creatine phosphate and adenosine diphosphate through a series of subsequent reactions to produce NADPH, the concentration of which is measured colorimetrically. The absorbance change is directly proportional to the CK activity in the sample [14]. The dynamic range of the CK assay on this platform was 5 to 5000 U/L. Values >5000 U/L could not be accurately quantified and were expressed as “>5000 U/L.” In the analysis, a value of 5000 U/L was assigned to these measurements. As a rule, the biochemical panel, including CK, was obtained daily from patients in the EVD treatment unit.

The viral load was determined by the Xpert Ebola assay (Cepheid) [11]. The nucleoprotein crossing threshold (Ct) from the reverse transcription–polymerase chain reaction was used as a surrogate of viral load.

### Clinical Definitions

Elevated CK was defined as CK >380 U/L [8]. Rhabdomyolysis was defined as a CK level >1000 U/L [8]. Severe rhabdomyolysis was defined as CK ≥5000 U/L [15].

AKI was defined per the KDIGO guidelines (Kidney Disease: Improving Global Outcomes) [16]. Stage 1 AKI was defined as an increase in creatinine (Cr) by ≥26.5 µmol/L within 48 hours or an increase in Cr to ≥1.5× baseline that is known or presumed to have occurred within the prior 7 days. Stage 2 was defined as an increase in Cr to *≥*2× to 2.9× baseline. Stage 3 was defined as an increase in Cr to *≥*3× baseline or *≥*353.6 mmol/L or, in patients aged <18 years, a decrease in estimated glomerular filtration rate to <35 mL/min/1.73 m^2^. To calculate the baseline Cr, we assumed a baseline glomerular filtration rate of 120 mL/min/1.73 m^2^ for children (<18 years of age), 100 mL/min/1.73 m^2^ in young adults (≥18 and <40 years), and 100 × 0.988^(age – 40)^ mL/min/1.73 m^2^ in older adults (≥40 years) [17]. We used the Full Age Spectrum–Age equation to back calculate the baseline Cr [18].

Liver injury was defined as alanine aminotransferase >240 IU/L (approximately 5 times the upper limit of normal) [19]. Elevated viral load was defined as nucleoprotein Ct <20 [19]. Vaccination status with the recombinant vesicular stomatitis virus–Zaire EBOV (Ervebo) was self-reported or reported by an accompanying family member.

### Statistical Analysis

Data analyses were performed with R software (version 3.2.6) [17]. Data visualization was based on Prism version 6 (GraphPad Software Inc). Descriptive statistics included the number and percentage for binary and categorical variables and the median and IQR for continuous variables. To examine associations among variables, nonparametric methods (Mann-Whitney *U* test) were used for continuous data, and the 2-tailed Pearson chi-square or Fisher exact test was used for categorical data, as appropriate. Longitudinal analysis of CK levels was conducted per a linear mixed effects (LME) regression model. Further details of the model are described in the supplementary methods (supplementary appendix). Bivariable and multivariable logistic regression models were used to confirm the associations among CK, AKI, and mortality, with adjustment for covariates. Statistical analysis of mortality was performed through bivariable and multivariable Cox proportional hazard models. We followed the method of Baron and Kenny [20] to perform a mediation analysis of AKI as a mediator of the association between rhabdomyolysis and death. Further details of the mediation analysis are provided in the supplementary methods (supplementary appendix).

Rhabdomyolysis and AKI were alternatively conceptualized as predictor variables and outcomes in different analyses. For our first objective (risk factors for rhabdomyolysis), variables measurable at admission to the EVD treatment unit were assessed. In this analysis, rhabdomyolysis was the outcome and was defined as 1 or more measurements of CK >1000 U/L over the course of hospitalization. For our second objective (predictors of AKI), rhabdomyolysis was a predictor variable, defined as admission CK >1000 U/L, and AKI was the outcome, defined as 1 or more Cr measurements meeting criteria for stage 1, 2, or 3 AKI over the course of hospitalization. For our third objective (predictors of mortality), rhabdomyolysis and AKI were predictor variables, defined by admission CK >1000 U/L and admission Cr meeting criteria for AKI, respectively.

### Ethics Approval

This study was made possible thanks to the EVD outbreak response conducted under the responsibility of the Secrétariat Technique du Comité Multisectoriel de Lutte contre la Maladie à Virus Ebola, itself under the supervision of the presidency of the DRC. The study was approved by the Comité d’Éthique du Nord Kivu (Centre Hospitalier Universitaire du Graben, Butembo, DRC) and the University of Alberta Human Research Ethics Board (Pro00095910). Informed consent was waived for this retrospective chart review.

## RESULTS

**Figure 1.**
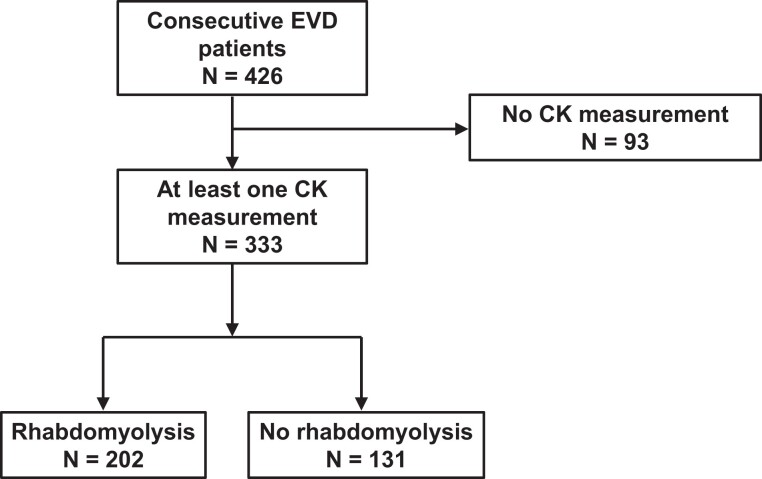
Trial profile. Of 426 consecutive patients admitted to the treatment units in Butembo and Katwa, Democratic Republic of the Congo, between 30 March and 1 October 2020, 93 had no CK measurement and were not included in subsequent analysis. CK, creatine kinase; EVD, Ebola virus disease.

From 30 March to 1 October 2019, EVD treatment units in Butembo and Katwa admitted 426 patients with confirmed EVD. Ninety-three patients (22%) had no measurement of CK and were excluded from the current analysis (Supplementary Table 1). We retrieved 2229 CK measurements from the remaining 333 patients (measurements per patient: median, 5; IQR, 1–11). Characteristics of these patients are shown in Table 1. Over the course of the admission, 271 (81%) patients had at least 1 elevated CK level (>380 U/L), 202 (61%) had rhabdomyolysis (CK >1000 IU/L), and 45 (14%) had severe rhabdomyolysis (≥5000 U/L). The trial flow diagram is shown in Figure 1.

**Table 1.** Characteristics of Patients With EVD Stratified by Rhabdomyolysis

	Overall (n = 333)	Rhabdomyolysis^a^ (n = 202)	No Rhabdomyolysis (n = 131)	*P* Value
Age, y	30 (20–46)	31 (20–47)	30 (20–45)	.57
<18	69 (21)	42 (61)	27 (39)	
18–40	156 (47)	94 (60)	62 (40)	
>40	108 (32)	66 (61)	42 (39)	
Sex				.024
Male	141 (42)	96 (68)	45 (32)	
Female	192 (58)	106 (55)	86 (45)	
Treatment center				<.0001
Butembo	235 (71)	160 (68)	75 (32)	
Katwa	98 (29)	42 (43)	56 (57)	
Known EVD contact	231 (69)	133 (58)	98 (42)	.11
Time from symptom onset to admission, d	4 (2–6)	4 (3–6)	3 (2–5)	.0031
Prior vaccination with rVSV-ZEBOV^b^	74 (22)	41 (55)	33 (45)	.36
Nucleoprotein Ct				
Admission	23.2 (20–27.4)	22.3 (19.8–26.1)	27.0 (23.0–32.0)	<.0001
Peak	22.1 (19.4–26.6)	21.1 (19.3–24.6)	26.2 (22.2–31.4)	<.0001
Antiviral treatment				
ZMapp	44 (13)	31 (70)	13 (30)	.21
Atoltivimab/maftivimab/odesivimab	63 (19)	44 (70)	19 (30)	.13
Ansuvimab	69 (21)	45 (65)	24 (35)	.46
Remdesivir	40 (12)	29 (73)	11 (27)	.14
None	9 (2.7)	6 (67)	3 (33)	>.99
Missing	108 (32)	47 (44)	61 (56)	<.0001
AKI^c^				<.0001^d^
None	119 (39)	56 (47)	63 (53)	
Stage 1	59 (19)	34 (58)	25 (42)	
Stage 2	25 (8.1)	17 (68)	8 (32)	
Stage 3	104 (34)	87 (84)	17 (16)	
Liver injury				
Peak AST, IU/L	560 (200–1300)	960 (350–1800)	230 (100–710)	<.0001
Peak ALT, IU/L	250 (130–450)	340 (190–590)	140 (68–270)	<.0001
Peak bilirubin, µmol/L	0.8 (0.6–1.2)	0.9 (0.7–1.5)	0.7 (0.6–0.9)	<.0001
Outcome^e^				
Fatal	118 (36)	98 (83)	20 (17)	<.0001
Survived	214 (64)	104 (49)	110 (51)	
Duration of hospitalization, d^f^	16 (14–19)	17 (15–20)	16 (12–18)	.033

Data are presented as median (IRQ) or No. (%)

Abbreviations: AKI, acute kidney injury; ALT, alanine aminotransferase; AST, aspartate aminotransferase; CK, creatine kinase; Cr, creatinine; Ct, crossing threshold; EVD, Ebola virus disease; rVSV-ZEBOV, recombinant vesicular stomatitis virus–Zaire Ebola virus.

^a^Rhabdomyolysis was defined as 1 or more measurements of CK >1000 U/L.

^b^Vaccination status was ascertained by self-report or report of a family member.

^c^AKI stage was defined as the highest stage during hospitalization and was based on all available Cr measurements. AKI was missing in 26 patients.

^d^ *P* value for any stage AKI vs no AKI.

^e^Vital status (outcome) was missing in 1 patient.

^f^Among survivors.

### Longitudinal Profile of CK in Patients With EVD

**Figure 2.**
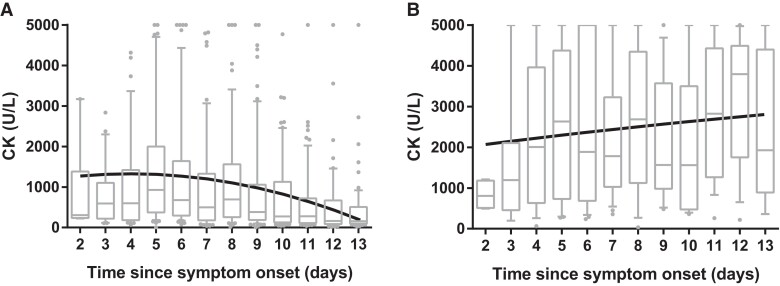
Longitudinal course of CK in patients with Ebola virus disease. *A*, Survivors had peak CK levels 3 days after symptom onset, with a subsequent decline. *B*, Fatal cases showed a monotonic increase in CK levels until death. Gray box plots show each day after symptom onset; the black line represents a linear mixed effects model of CK as a quadratic function of time (supplementary materials). Median, line; IQR, box; 95% CI, error bars; circle, outlier. CK, creatine kinase.

In patients admitted to the EVD treatment units who survived, the median CK level was 550 U/L (IQR, 200–1500) at admission. The peak CK occurred at day 3.4 postadmission (IQR, 2.3–5.5) at a median level of 1600 U/L (IQR, 550–3400) and decreased thereafter (Figure 2*A*). In fatal cases (Figure 2*B*), the median CK level at admission was significantly higher than among survivors (1900 U/L; IQR, 660–3400; *P* < .0001). In fatal cases, the CK levels increased until death, with a median peak recorded at 2900 U/L (IQR, 1500–4900), comparably higher than that observed in survivors (*P* < .0001). The longitudinal trajectory of CK, modeled as a function of time via a LME model, showed striking differences between survivors and nonsurvivors (Figure 2, Supplementary Table 2).

### Predictors of Subsequent Rhabdomyolysis: Present at Admission to the EVD Treatment Unit

Male sex, longer time from onset of symptoms to admission, and lower Ct at admission were associated with subsequent rhabdomyolysis during hospitalization in a bivariable analysis (Supplementary Table 3). Male sex and Ct <20 remained statistically significant independent predictors of rhabdomyolysis in a multivariable model adjusting for covariates.

### Association of Rhabdomyolysis With Kidney Injury

**Figure 3.**
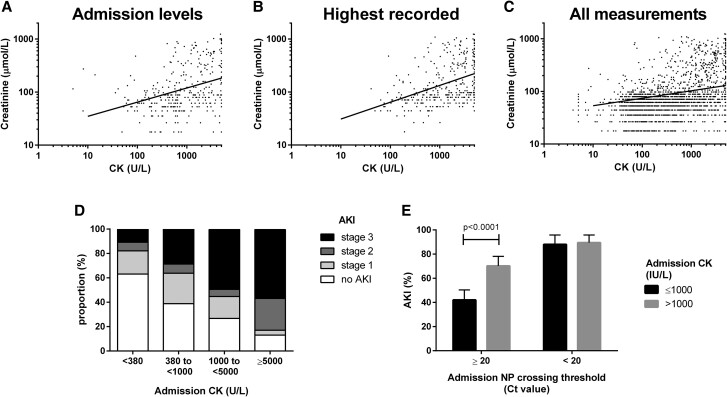
Rhabdomyolysis is associated with AKI. *A*, Cr and CK levels at admission were positively correlated (ρ = 0.40, *P* < .0001). *B*, Peak Cr levels (highest recorded Cr value for a patient during hospitalization) and peak CK levels (highest recorded CK value for the same patient during hospitalization) were positively correlated (ρ = 0.42, *P* < .0001). *C*, Sample-paired longitudinal measurements were significantly associated (*P* < .0001) in a linear mixed effects model of Cr as a function of CK, accounting for repeated measurements in patients. *D*, Increasing admission CK level was associated with increasing risk and stage of AKI during hospitalization, suggesting a dose-response effect. *E*, Confounding effects of viral load (surrogate: nucleoprotein polymerase chain reaction Ct) were addressed in a stratified analysis, demonstrating the independent effect of rhabdomyolysis on AKI in patients with low viral load (corresponding to Ct ≥20). In this analysis, Ct and rhabdomyolysis were predictor variables, as measured at admission. AKI was assessed by using all Cr measurements during hospitalization and taking the highest AKI stage. AKI, acute kidney injury; CK, creatine kinase; Cr, creatinine; Ct, crossing threshold; NP, nucleoprotein.

Rhabdomyolysis was associated with AKI (Table 1). A positive correlation was observed between CK and Cr at admission (Figure 3*A*; ρ = 0.40, *P* < .0001). The peak CK and peak Cr for each patient were correlated (Figure 3*B*; ρ = 0.42, *P* < .0001). Sample-paired values of CK and Cr measured longitudinally over the course of illness were positively correlated in a LME model (Figure 3*C*; *P* < .0001). There was a dose-response effect of increasing frequency and severity of AKI during hospitalization with increasing admission CK levels (Figure 3*D*). There was also a temporal relationship: elevated CK at admission was associated with subsequent deterioration of kidney function, defined as a rise in Cr >26.5 mmol/L within 48 hours (odds ratio [OR], 2.4; 95% CI, 1.1–5.9; *P* = .033) [21]. Lower reverse transcription–polymerase chain reaction nucleoprotein Ct (higher viral load) at admission was associated with rhabdomyolysis and AKI (potential confounder); therefore, we performed a stratified analysis to determine the association between rhabdomyolysis at admission and AKI during hospitalization at different Ct levels. We found that rhabdomyolysis at admission was associated with higher risk of AKI during hospitalization in patients with high Ct but not low Ct (Figure 3*E*). Finally, in a multivariable model adjusting for covariates, rhabdomyolysis at admission remained an independent predictor of AKI during hospitalization (adjusted OR, 1.8; 95% CI, 1.0–3.3; *P* = .045; Supplementary Table 4).

### Rhabdomyolysis at Admission Is an Independent Predictor of Mortality

**Figure 4.**
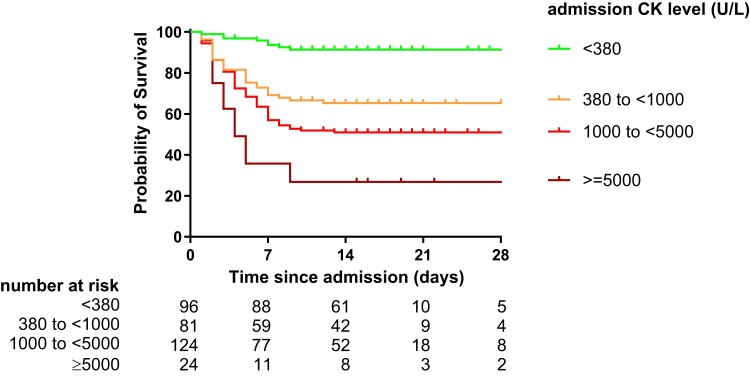
Rhabdomyolysis is associated with increased mortality. Increasing levels of CK at admission were associated with shorter time to death. The hazard of death among patients with rhabdomyolysis at admission was 3.1 fold higher (95% CI, 2.1–4.7) than patients without rhabdomyolysis (*P* < .0001). CK, creatine kinase.

Survival analysis demonstrated a shorter time to death among patients with rhabdomyolysis at admission (Supplementary Table 5). The survival curves showed a dose-response relationship with increasing admission CK levels (Figure 4). In a multivariable Cox proportional hazard model adjusting for covariates, including AKI, rhabdomyolysis at admission remained a statistically significant independent predictor of mortality (adjusted hazard ratio, 1.7; 95% CI, 1.03–2.9; *P* = .037).

### AKI Partially Mediates the Effect of Rhabdomyolysis on Mortality

A directed acyclic graph [22] representing the complex relationship among rhabdomyolysis, AKI, and mortality is shown in Supplementary Figure 1. In particular, biological considerations suggested that AKI may mediate the association between rhabdomyolysis and mortality. The association between rhabdomyolysis and mortality (odds ratio, 4.0; 95% CI, 2.5–6.5; Supplementary Table 6) was significantly reduced when AKI was included as an independent variable in the model (OR, 2.6; 95% CI, 1.5–4.3), suggesting its role as a mediator variable (proportion mediated, 0.32; 95% CI, .16–.55; *P* < .0001). We concluded that AKI partially mediated the effect of rhabdomyolysis on mortality.

## DISCUSSION

Here we describe a case series of 333 EVD patients in whom CK was measured. Noteworthy findings were the association of (1) lower Ct (higher viral load) with rhabdomyolysis, (2) rhabdomyolysis with AKI, and (3) rhabdomyolysis with mortality.

Rhabdomyolysis has been recognized as a complication of several viral diseases [8], including EVD. Clinical observations of myalgia were described as early as the first Ebola disease outbreak in Sudan in 1976 [23]. Biochemical evidence of myocyte injury (elevated CK) has been reported in several previous studies of patients with EVD [8, 19, 24], including an association with AKI. The median peak of CK was 1600 (IQR, 550–3400) in our patients, which is similar to the peak levels of 1900 U/L (IQR, 590–3400) in a study from Sierra Leone [19]. In our study, 61% of our patients had rhabdomyolysis and 14% had severe rhabdomyolysis (CK ≥5000 U/L), which is similar to the respective rates of 59% and 36% in patients from a Guinean study (2014–2016) [8].

The mechanisms of EVD-associated rhabdomyolysis remain incompletely understood. Rhabdomyolysis is characterized by acute damage to the myocyte sarcolemma, leading to release of toxins into the circulation, such as myoglobin, electrolytes, and CK [9, 11]. Hypothesized mechanisms of virus-induced muscle damage include direct viral invasion of myocytes [25], immune-mediated damage by myotoxic cytokines [11, 26–30], and nitric oxide [31, 32]. The mechanism of direct EBOV invasion of myocytes has been investigated in experimental model systems. In a guinea pig disease model, EBOV was detected in the smooth muscle of the uterine wall, rarely in cardiomyocytes, but never in skeletal muscle [25]. Neither direct viral invasion of skeletal myocytes nor widespread muscle injury is observed in the nonhuman primate disease model [7]. This apparent discrepancy between animal models and biochemical abnormalities in human patients supports the need for further study of the source of the elevated CK in humans and the mechanism of myocyte injury. The higher EBOV viral load associated with rhabdomyolysis in our study may be consistent with direct viral cytopathy; however, indirect consequences of severe disease cannot be excluded as causes of the elevated CK.

Rhabdomyolysis due to EVD [24] or other pathologies [11] can lead to AKI. Our results showed a significant association between rhabdomyolysis and AKI (OR, 1.8). Although our observational study cannot demonstrate causality, this association met several criteria that suggested a causal link: temporality, dose-response, strength of association, coherence, and biological plausibility [33]. Previous authors suggested that rhabdomyolysis might play a role in the development of renal failure in EVD or Sudan virus disease; however, their sample sizes were too small to demonstrate a statistically significant association [8, 34, 35].

Rhabdomyolysis causes AKI by several mechanisms. Fluid sequestration within the damaged muscle leads to hypovolemia [36, 37] and decreased kidney perfusion. Free myoglobin induces intrarenal vasoconstriction, direct tubule injury, tubular obstruction, and macrophage-dependent inflammation [11, 38, 39]. Massive myocyte lysis is required to generate these insults such that AKI typically occurs at CK levels ≥15 000 U/L [11, 12, 37] when rhabdomyolysis is the sole cause of AKI. However, AKI may occur with CK ≥5000 U/L when associated with coexisting conditions (eg, sepsis, dehydration, and acidosis) [11]. In our study, AKI was observed at much lower CK levels: >380 U/L (61%), 1000 to <5000 (74%), and ≥5000 (87%). Our observations suggest that concomitant factors besides rhabdomyolysis contribute to renal damage in EVD. These might include gastrointestinal fluid loss [40], inflammatory response, and direct viral invasion of kidney cells leading to coagulative necrosis [41].

Rhabdomyolysis was associated with increased mortality in our study (adjusted hazard ratio, 1.7), as previously described [8, 19, 24]. Based on theory and consistent with our observations (Figure 3), there may be a causal link between rhabdomyolysis and death; furthermore, AKI may be an intermediate variable on this pathway. A mediation analysis suggested that 32% of the observed effect of rhabdomyolysis on mortality was mediated by AKI. Taken together, our results suggested that rhabdomyolysis contributed to mortality in part through AKI and in part through other mechanisms. Of note, our study is the first to include a sufficient number of patients with laboratory parameters to perform this multivariable modeling [8]. The complex relationship among rhabdomyolysis, AKI, and death is illustrated in Supplementary Figure 1. Additional complications of rhabdomyolysis, such as metabolic acidosis and hyperkalemia, may influence clinical outcome but were not included in our analysis.

In addition to AKI and mortality, rhabdomyolysis was associated with several other patient characteristics (Table 1). Rhabdomyolysis was more common among male patients, likely because of higher lean muscle mass leading to higher CK levels. The time from symptom onset to admission was longer in patients with rhabdomyolysis, which may indicate that delay in presentation is associated with more severe disease and muscle injury. The Ct was lower at admission in patients with rhabdomyolysis, corresponding to a higher viral load, which may suggest a direct or indirect viral effects. Liver transaminases and bilirubin were elevated in patients with rhabdomyolysis, consistent with a multiorgan dysfunction syndrome in which liver and skeletal muscle injury occurs concurrently in severe cases.

The EVD treatment unit (Butembo or Katwa) was a confounding factor associated with the frequency of rhabdomyolysis and AKI (Table 1, Supplementary Tables 3 and 4). The Katwa EVD treatment unit was a “transit center” that nonetheless admitted and managed confirmed cases of EVD. The most severe EVD cases in the area were generally directed to the Butembo EVD treatment unit. This management practice explains why cases at Katwa had lower rates of rhabdomyolysis and AKI than those at Butembo. We adjusted for site (Butembo or Katwa) as a potential confounding variable in the associations between rhabdomyolysis and AKI.

This retrospective observational study design has inherent limitations, making it difficult to draw causal inferences among rhabdomyolysis, AKI, and mortality. Nonetheless, we found strong associations, consistent with a mechanistic link between rhabdomyolysis and AKI, well recognized in other conditions. Additional studies are needed to clearly demonstrate the remaining criteria: consistency and mechanism (eg, experimental animal models). Twenty-two percent of patients with EVD were excluded from the study due to the lack of CK measurement, as death occurred rapidly after their admission (Supplementary Table 1). Patients with EVD in our study differed significantly from excluded patients in terms of baseline characteristics (eg, lower mortality, lower viral load). Therefore, our cohort is not representative of all admissions to the EVD treatment unit, and our results should be extrapolated with caution to patients who are the sickest. Due to the retrospective nature of our study, some parameters could not be retrieved from patients’ medical records over the course of their stay at EVD treatment units. The upper limit of the dynamic range of the Piccolo Xpress Chemistry Analyzer for CK was 5000 U/L. CK values >5000 U/L could not be quantified accurately. In the future, the use of truly quantitative CK assays over a wider dynamic range could further clarify the relationship among CK elevation, AKI, and outcome in EVD. Sex differences in CK reference ranges should be taken into account in future analyses. Furthermore, the lack of defined reference ranges for CK in the Congolese population limits the interpretation of our findings. Classification of AKI assumed a normal baseline glomerular filtration rate based on age. Documentation of each individual's glomerular filtration rate prior to disease onset would be desirable for accurate classification of AKI stage, though this would be challenging in practice. Additional data on volume status, acidosis, and electrolyte abnormalities would be helpful to understand other causes and consequences of AKI.

In summary, detailed longitudinal laboratory measurements on a large cohort of patients with EVD allowed us to perform multivariable modeling associating (1) viral load with rhabdomyolysis, (2) rhabdomyolysis with AKI, and (3) rhabdomyolysis with mortality. The conclusions are sufficiently strong to inform or reinforce clinical practice in the EVD treatment unit. CK and kidney function should be monitored to anticipate AKI and implement appropriate treatment (eg, intravenous fluids) [42]. Expectant management of rhabdomyolysis and AKI in EVD may contribute to improved mortality in this frequently lethal infection.

## Supplementary Data

Supplementary materials are available at *The Journal of Infectious Diseases* online (http://jid.oxfordjournals.org/). Supplementary materials consist of data provided by the author that are published to benefit the reader. The posted materials are not copyedited. The contents of all supplementary data are the sole responsibility of the authors. Questions or messages regarding errors should be addressed to the author.

## Supplementary Material

jiae224_Supplementary_Data

## Notes

***Disclaimer***. The funders had no role in study design, data collection, data analysis, data interpretation, writing of the report, or decision to submit the article for publication.

***Financial support*.** This was a nonfunded retrospective analysis of patients admitted to 2 EVD treatment units, which were part of the EVD outbreak response, conducted under the direction of the Secrétariat Technique du Comité Multisectoriel de Lutte contre la Maladie à Virus Ebola, itself under the supervision of the presidency of the DRC. Laboratory supplies and equipment used for patient diagnostics and care were made available to the Institut National de Recherche Biomédicale by international partners, including the US National Institutes of Health and the World Health Organization.
