# Supplementary material for: Rhabdomyolysis, Acute Kidney Injury, and Mortality in Ebola Virus Disease: Retrospective Analysis of Cases From the Eastern Democratic Republic of the Congo, 2019
Source: J Infect Dis. 2024 May 2;230(2):e465–73. doi: 10.1093/infdis/jiae224 (PMC11326845; doi:10.1093/infdis/jiae224)
Supplement: jiae224_Supplementary_Data [file jiae224_supplementary_data.docx]

**Supplemental Methods**

*Longitudinal CK levels in fatal and non-fatal EVD cases*

To model the CK over time, we followed the method of Lanini et. al., who previously described the kinetics of viremia after infection by Ebola virus (EBOV) using statistical modeling [1]. CK as a function of time after symptom onset was modeled with a quadratic maximum likelihood multilevel linear mixed effects regression model. The model included random intercept at patient level and random slope at time level [1]. Previous studies have employed these models, referred as growth curve models or latent trajectory models [2], to analyze data in which repeated measures are taken over time (e.g., clustered longitudinal data). As in the previous study, we restricted the analysis to days 2-13 after symptom onset [1].

Random intercept and random slope were assessed using the likelihood ratio test (LRT). The random intercept was included if LRT p-value ≤ 0.1, comparing a standard null model (i.e., the model including only the dependent variable) *versus* the random intercept null model. The random slope was included if LRT p-value ≤ 0.1, comparing the random intercept null model *versus* the random intercept plus the random slope null model. Previous authors have shown that EBOV viremia is a quadratic function of time, reaching a peak at 5 to 7 days after symptom onset, and declining thereafter in patients who survive the infection [1]. We hypothesized that CK would also follow a similar trajectory, based on unadjusted boxplots of CK as a function of time (Figure 2). We verified the functional form of association between EBOV viremia and time using LRT to compare models with CK (dependent variable) and time since symptom onset (independent variable) as a continuous variable with linear and quadratic terms. Interaction between mortality and time was assessed by LRT and were included if LRT p-value ≤ 0.1.

*Mediation analysis*

We followed the method of Baron and Kenny [3] to perform a mediation analysis of AKI as a mediator of the association between rhabdomyolysis and death, using the package *mediation* [4] in the R statistical environment. We set rhabdomyolysis as the exposure, in-hospital mortality as the outcome, and AKI as the mediator. Logistic regression models were fitted for the outcome variable and for the mediator variable. Our null hypothesis was that either or both of the rhabdomyolysis-AKI and AKI-mortality associations was zero. The total effect was partitioned into the average direct effect, which is the effect of the exposure not explained by the mediator, and the average causal mediation effect, which is the proportion of the effect of the exposure explained by the mediator and the interaction between the exposure and the mediator. The CIs were derived by bootstrapping based on 1000 replications.

**Supplemental Table S1. Characteristics of EVD patients, according to missing CK**

|  | **Overall**  **(N=426)** | **One or more CK measurements**  **(N=333)** | **No CK**  **measurement**  **(N=93)** | **P-value** |
| --- | --- | --- | --- | --- |
| **Age, median (IQR)** | 30 (20-45) | 30 (20-46) | 30 (21-44) | 0.96 |
| **<18 years, n (%)** | 82 (19) | 69 (21) | 13 (14) |  |
| **18-40, n (%)** | 209 (49) | 156 (47) | 53 (58) |  |
| **>40, n (%)** | 134 (32) | 108 (32) | 26 (28) |  |
| **Sex, n (%)** |  |  |  | 0.23 |
| **Male** | 187 (44) | 141 (42) | 46 (50) |  |
| **Female** | 238 (56) | 192 (58) | 46 (50) |  |
| **Treatment Centre, n (%)** |  |  |  | 0.00097 |
| **Butembo** | 283 (66) | 235 (71) | 48 (52) |  |
| **Katwa** | 143 (34) | 98 (29) | 45 (48) |  |
| **Known EVD contact, n (%)** | 275 (65) | 231 (69) | 44 (47) | 0.00014 |
| **Time from symptom onset to admission [days], median (range)** | 4 (2-6) | 4 (2-6) | 5 (3-7) | 0.0089 |
| **Prior vaccination with rVSV-ZEBOV**^a^ | 79 (19) | 74 (22) | 5 (5.4) | <0.0001 |
| **Nucleoprotein crossing threshold (Ct)** |  |  |  |  |
| **Admission** | 23.2 (20.0-27.4) | 24.1 (20.5-28.2) | 22.8 (19.9-27.6) | <0.0001 |
| **Nadir** | 22.1 (19.4-26.6) | 22.8 (19.9-27.6) | 20.4 (18.0-22.9) | <0.0001 |
| **Treatment** |  |  |  |  |
| **ZMapp** | 49 (12) | 44 (13) | 5 (5.4) | 0.056 |
| **Atoltivimab/maftivimab/odesivimab** | 68 (16) | 63 (19) | 5 (5.4) | 0.0028 |
| **Ansuvimab** | 75 (18) | 69 (21) | 6 (6.5) | 0.0024 |
| **Remdesivir** | 47 (11) | 40 (12) | 7 (7.5) | 0.30 |
| **None** | 38 (8.9) | 9 (2.7) | 29 (31) | <0.0001 |
| **Missing** | 149 (35) | 108 (32) | 41 (44) | 0.050 |
| **Acute kidney injury (AKI)** |  |  |  | 0.29^b^ |
| **None** | 121 (38) | 119 (39) | 2 (18) |  |
| **Stage 1** | 60 (19) | 59 (19) | 1 (9.1) |  |
| **Stage 2** | 25 (7.9) | 25 (8.1) | 8 (73) |  |
| **Stage 3** | 112 (35) | 104 (34) | 2 (18) |  |
| **Liver injury** |  |  |  |  |
| **Peak AST [IU/L], median (IQR)** | 550 (190-1300) | 560 (200-1300) | 170 (52-300) | 0.018 |
| **Peak ALT [IU/L], median (IQR)** | 250 (120-450) | 250 (130-450) | 290 (52-420) | 0.88 |
| **Peak bilirubin [µmol/L], median (IQR)** | 0.8 (0.6-1.2) | 0.8 (0.6-1.2) | 0.5 (0.5-1) | 0.12 |
| **Outcome** |  |  |  |  |
| **Fatal, n (%)** | 200 (48) | 120 (37) | 80 (86) | <0.0001 |
| **Survived, n (%)** | 219 (52) | 206 (63) | 13 (14) | <0.0001 |
| **Duration of hospitalization [days], median (range)**^c^ | 16 (13-19) | 16 (14-19) | 11 (8-17) | 0.022 |

^a^Vaccination status was ascertained by self-report or by report of a family member

^b^p-value for any stage AKI *versus* no AKI

^c^Among survivors

**Supplemental Table S2. Model parameters: kinetics of creatine kinase in EVD patients**

| **Overall model parameters** |  |  |  |
| --- | --- | --- | --- |
| Number of observations | 777 |  |  |
| Random intercept group variable: patients | 202 |  |  |
| Random slope variable (time): days | 2-13 |  |  |
| Observations per patient, median (range) | 3 (1-6) |  |  |
| **Model selection** |  |  |  |
| *Random effect (assessed on null model)* |  |  |  |
| Random intercept vs. standard linear regression model | p<0.001 |  |  |
| Random slope vs random intercept model | p<0.001 |  |  |
| *Functional form of association between CK and time* |  |  |  |
| Linear vs quadratic | p<0.001 |  |  |
| Interaction between time and mortality vs no interaction | p<0.001 |  |  |
| **Fixed effect parameters** | **Coeff.** | **95% CI** | **p-value** |
| Time after symptom onset: linear | 110 | -42 to 260 | <0.001^a^ |
| Time after symptom onset: quadratic | -14 | -22 to -5.4 | 0.0059^b^ |
| Mortality | 720 | -860 to 2300 | <0.001^c^ |
| *Interaction* |  |  |  |
| Time after symptom onset (linear) and mortality | -28 | -400 to 340 |  |
| Time after symptom onset (quadratic) and mortality | 13 | -9.5 to 35 | 0.00027^d^ |
| Constant: Intercept at Time after symptom onset=0 | 1200 | 430 to 1900 |  |
| **Random effect parameters** | **Coeff.** | **95% CI** |  |
| Random slope | 170 | 130 to 210 |  |
| Random intercept | 2200 | 1800 to 2500 |  |
| Residual variance | 760 | 710 to 810 |  |

^a^Model with linear time parameter *versus* model without time

^b^Full model *versus* model without quadratic time parameter

^c^Full model *versus* model without mortality parameter

^d^Full model *versus* model without time-mortality interaction parameters

**Supplemental Table S3. Risk factors, determined at admission, for rhabdomyolysis: bivariable and multivariable logistic regression models**

| **Independent variable** | **OR**^a^ | **P-value** | **aOR**^b^ | **P-value** |
| --- | --- | --- | --- | --- |
| **Age** | 1 (0.99-1) | 0.56 | 1 (1-1) | 0.07 |
| **Sex** |  |  |  |  |
| **Male** | 1.0 (reference) |  | 1.0 (reference) |  |
| **Female** | 0.58 (0.37-0.91) | 0.018 | 0.43 (0.25-0.72) | 0.0014 |
| **Site** |  |  |  |  |
| **Butembo** | 1.0 (reference) |  | 1.0 (reference) |  |
| **Katwa** | 0.35 (0.22-0.57) | <0.0001 | 0.62 (0.13-2.8) | 0.53 |
| **Time from symptom onset to admission** |  |  |  |  |
| **<4 days** | 1.0 (reference) |  | 1.0 (reference) |  |
| **≥4 days** | 1.6 (1-2.6) | 0.032 | 1.5 (0.9-2.4) | 0.13 |
| **Admission NP crossing threshold (Ct)** |  |  |  |  |
| **≥ 20** | 1.0 (reference) |  | 1.0 (reference) |  |
| **< 20** | 2.9 (1.6-5.7) | 0.001 | 3.1 (1.6-6.4) | 0.00097 |
| **Treatment** |  |  |  |  |
| **ZMapp** | 1.0 (reference) |  | 1.0 (reference) |  |
| **Atoltivimab/maftivimab/odesivimab** | 0.97 (0.41-2.2) | 0.95 | 1.1 (0.47-2.8) | 0.77 |
| **Ansuvimab** | 0.79 (0.34-1.8) | 0.56 | 0.93 (0.39-2.2) | 0.87 |
| **Remdesivir** | 1.1 (0.43-2.9) | 0.84 | 1.2 (0.44-3.2) | 0.74 |
| **None** | 0.84 (0.19-4.4) | 0.82 | 0.95 (0.2-5.3) | 0.95 |

NP, nucleoprotein

^a^From bivariable models

^b^From multivariable model

**Supplemental Table S4. Risk factors, determined at admission, for acute kidney injury during hospitalization: bivariable and multivariable logistic regression models**

| **Independent variable^a^** | **OR**^a^ | **P-value** | **aOR**^b^ | **P-value** |
| --- | --- | --- | --- | --- |
| **Rhabdomyolysis at admission** | 3.2 (2.0-5.3) | <0.0001 | 2.2 (1.2-3.8) | 0.0065 |
| **Age** |  |  |  |  |
| **<18 years, n (%)** | 0.4 (0.2-0.77) | 0.0079 | 1.7 (0.81-3.9) | 0.16 |
| **18-40, n (%)** | 1.0 (reference) |  | 1.0 (reference) |  |
| **>40, n (%)** | 0.35 (0.17-0.71) | 0.0041 | 1.1 (0.60-2.1) | 0.75 |
| **Sex, n (%)** |  |  |  |  |
| **Male** | 1.0 (reference) |  |  |  |
| **Female** | 0.99 (0.62-1.6) | 0.96 |  |  |
| **Treatment Centre, n (%)** |  |  |  |  |
| **Butembo** | 1.0 (reference) |  | 1.0 (reference) |  |
| **Katwa** | 0.33 (0.19-0.55) | <0.0001 | 0.39 (0.20-0.74) | 0.0044 |
| **Known EVD contact, n (%)** | 0.54 (0.32-0.91) | 0.023 | 0.59 (0.31-1.1) | 0.099 |
| **Time from symptom onset to admission >4 days** | 3.3 (2.0-5.6) | <0.0001 | 2.7 (1.5-4.9) | 0.0013 |
| **Prior vaccination with rVSV-ZEBOV**^c^ | 0.38 (0.22-0.64) | 0.00038 | 0.63 (0.32-1.2) | 0.19 |
| **Admission NP Ct < 20** | 6.8 (3.2-17) | <0.0001 | 5.7 (2.8-13) | <0.0001 |
| **Treatment** |  |  |  |  |
| **ZMapp** | 1.0 (reference) |  |  |  |
| **Atoltivimab/maftivimab/odesivimab** | 0.66 (0.28-1.5) | 0.33 |  |  |
| **Ansuvimab** | 1.4 (0.58-3.2) | 0.47 |  |  |
| **Remdesivir** | 0.74 (0.29-1.9) | 0.52 |  |  |
| **None** | ∞ (0.74-∞) | 0.09 |  |  |
| **Missing** | 0.34 (0.15-0.73) | 0.0067 |  |  |

NP, nucleoprotein; Ct, crossing threshold

^a^From bivariable models

^b^From multivariable model

^c^Vaccination status was ascertained by self-report or by report of a family member

**Supplemental Table S5. Risk factors, determined at admission, for mortality^a^: bivariable and multivariable logistic regression models**

|  | **HR (95% CI)** | **P-value** | **aHR (95% CI)** | **P-value** |
| --- | --- | --- | --- | --- |
| **Rhabdomyolysis at admission** | 3.7 (2.3-6.0) | <0.0001 | 1.7 (1.03-2.9) | 0.037 |
| **AKI at admission** | 5.2 (3.4-8.0) | <0.0001 | 2.2 (1.4-3.6) | 0.00090 |
| **Age** |  |  |  |  |
| **<18 years, n (%)** | 1.5 (0.96-2.4) | 0.077 |  |  |
| **18-40, n (%)** | 1.0 (reference) |  |  |  |
| **>40, n (%)** | 1.2 (0.78-1.8) | 0.42 |  |  |
| **Sex, n (%)** |  |  |  |  |
| **Male** | 1.0 (reference) |  |  |  |
| **Female** | 0.91 (0.63-1.3) | 0.63 |  |  |
| **Known EVD contact, n (%)** | 0.70 (0.48-1.0) | 0.063 |  |  |
| **Time from symptom onset to admission >4 days** | 1.8 (1.3-2.7) | 0.0011 | 1.1 (0.78-1.7) | 0.47 |
| **Prior vaccination with rVSV-ZEBOV**^b^ | 0.39 (0.22-0.70) | 0.0015 | 0.55 (0.30-1.0) | 0.055 |
| **Admission NP Ct < 20** | 8.1 (5.6-12) | <0.0001 | 3.9 (2.6-5.9) | <0.0001 |
| **Treatment** |  |  |  |  |
| **ZMapp** | 1.0 (reference) |  |  |  |
| **Atoltivimab/maftivimab/odesivimab** | 0.91 (0.47-1.8) | 0.78 |  |  |
| **Ansuvimab** | 0.93 (0.48-1.8) | 0.82 |  |  |
| **Remdesivir** | 1.3 (0.63-2.5) | 0.52 |  |  |
| **None** | 11 (4.7-28) | <0.0001 | 4.0 (1.9-8.7) | 0.00036 |
| **Missing** | 0.8 (0.44-1.5) | 0.48 |  |  |
| **Liver injury (ALT>240 IU/L)** | 5.5 (3.7-8.2) | <0.0001 | 2.1 (1.3-3.4) | 0.0014 |

NP, nucleoprotein; Ct, crossing threshold

^a^Missing mortality outcome in 4 patients.

^b^Vaccination status was ascertained by self-report or by report of a family member

**Supplemental Table S6. Mediation analysis: AKI as mediator of association between rhabdomyolysis and mortality**

|  | **OR (95% CI)** | **P-value** | **Model type** |
| --- | --- | --- | --- |
| *Model 1 (total effect)* |  |  | logistic regression |
| rhabdomyolysis → mortality | 4.0 (2.5-6.5) | <0.0001 |  |
| *Model 2* |  |  | logistic regression |
| rhabdomyolysis → AKI | 3.2 (2.0-5.3) | <0.0001 |  |
| *Model 3* | **aOR (95% CI)** |  | logistic regression |
| rhabdomyolysis + AKI → mortality |  |  |  |
| rhabdomyolysis | 2.6 (1.5-4.3) | 0.00046 |  |
| AKI | 5.9 (3.2-11) | <0.0001 |  |
| *Mediation analysis* | **Estimate (95%CI)** |  |  |
| Average causal mediation effect | 0.090 (0.043-0.14) | <0.0001 |  |
| Average direct effect | 0.18 (0.085-0.29) | <0.0001 |  |
| Total effect | 0.27 (0.17-0.37) | <0.0001 |  |
| Proportion Mediated | 0.32 (0.16-0.55) | <0.0001 |  |

In this analysis, rhabdomyolysis (at admission) is the exposure, mortality is the outcome, and AKI (during hospitalization) is the mediator.


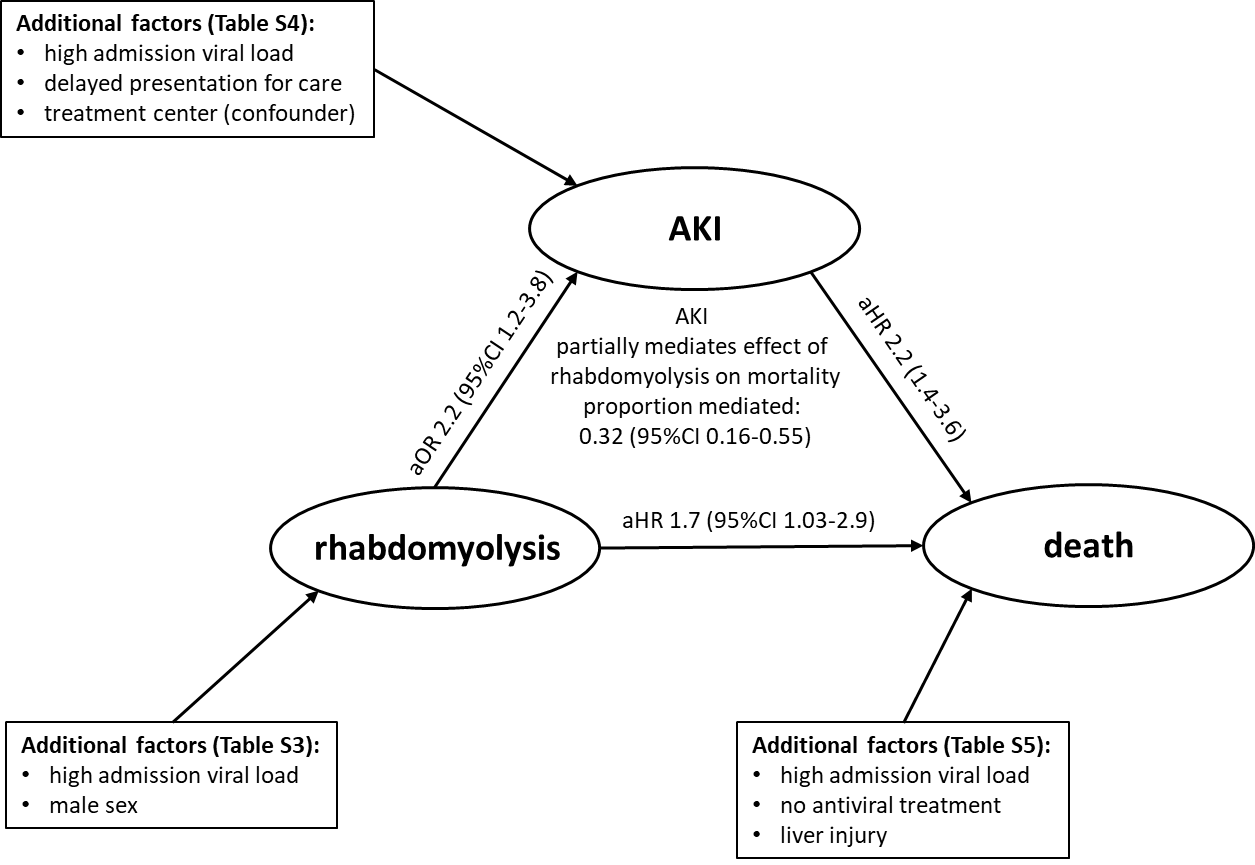


**Figure S1. Directed acyclic graph (DAG) of relationship between rhabdomyolysis, AKI, and mortality in the EVD cohort.** Several multivariable models were developed to identify the independent risk factors for rhabdomyolysis (Supplemental Table S3), AKI (Supplemental Table S4), and mortality (Supplemental Table S5). These are synthesized in the above DAG. Biological considerations suggested that AKI may mediate the association between rhabdomyolysis and mortality. In a mediation analysis, AKI partially mediated the effect of rhabdomyolysis on mortality.

**Supplemental references**

[1] Lanini S, Portella G, Vairo F, Kobinger GP, Pesenti A, Langer M, et al. Blood kinetics of Ebola virus in survivors and nonsurvivors. J Clin Invest. 2015;125:4692-8.

[2] Curran PJ, Hussong AM. The use of latent trajectory models in psychopathology research. J Abnorm Psychol. 2003;112:526-44.

[3] Baron RM, Kenny DA. The moderator-mediator variable distinction in social psychological research: conceptual, strategic, and statistical considerations. J Pers Soc Psychol. 1986;51:1173-82.

[4] Tingley D, Yamamoto HT, Hirose K, Keele L, Princeton KI. mediation: R Package for Causal Mediation Analysis. <http://CRAN.R-project.org/package=mediation> (accessed 6 Sept 2023).
